# Incidental Gonadal Vein Thrombosis Diagnosed Using Computed Tomography Imaging: A Single-Center, Retrospective, Cohort Study

**DOI:** 10.7759/cureus.15741

**Published:** 2021-06-18

**Authors:** Shaza Alsharif, Ahmed Subahi, Bader Shirah, Khalid M Alshamrani, Turki A Alhazmi, Benoit Mesurolle

**Affiliations:** 1 Medical Imaging, King Abdullah International Medical Research Center, Jeddah, SAU; 2 Medical Imaging, King Saud Bin Abdulaziz University for Health Sciences, Jeddah, SAU; 3 Medical Imaging, Ministry of the National Guard - Health Affairs, Jeddah, SAU; 4 College of Science and Health Professions, King Saud Bin Abdulaziz University for Health Sciences, Jeddah, SAU; 5 Research Office, King Abdullah International Medical Research Center, Jeddah, SAU; 6 College of Medicine, King Saud Bin Abdulaziz University for Health Sciences, Jeddah, SAU; 7 College of Applied Medical Sciences, King Saud Bin Abdulaziz University for Health Sciences, Jeddah, SAU; 8 College of Medicine, Umm Alqura University, Makkah, SAU; 9 Department of Radiology, Pôle Santé République, Clermont-Ferrand, FRA

**Keywords:** ovarian vein, gonadal vein, thrombosis, computed tomography, imaging

## Abstract

Objectives

Gonadal vein thrombosis is an uncommon but serious condition that can be fatal if it goes unnoticed. Up to 80% of cases occur in patients after delivery, hysterectomy, or lymphadenectomy for gynecological neoplasms. The objective of this study was to determine the incidence of gonadal vein thrombosis using computed tomography (CT) imaging at our center and to describe associated risk factors.

Methods

A retrospective, single-center, observational study was conducted at King Abdulaziz Medical City in Jeddah, Saudi Arabia. Data were collected for all patients diagnosed with incidental gonadal-vein-thrombosis using contrast-enhanced computed tomography imaging of the abdomen and pelvis between January 2005 and December 2017. We included all patients with incidental findings of gonadal vein thrombosis and excluded those with incomplete data.

Results

In total, 58/68,268 (0.08%) patients were included. Fifty-seven patients were females, and only one was male. The mean age (years) of the patients was 50.0±15.0 (range 4-87). Thirty-four patients (59%) had right gonadal vein thrombosis, 20 (34%) had thrombosis on the left side, and four (7%) had bilateral thrombosis. Nineteen patients (33%) had undergone pelvic surgery previously. Forty-four patients (76%) had a malignancy at the time of diagnosis. Forty-two patients (72%) were treated with anticoagulants after the diagnosis.

Conclusion

Gonadal vein thrombosis is a rare clinical entity with vague clinical presentation. The incidence in the oncology population is similar to the reported incidence in the obstetric population. Initiation of anticoagulation therapy is important to treat this condition and limit complications.

## Introduction

Gonadal vein thrombosis (GVT) is an exceedingly uncommon but serious condition that can be fatal if it goes unnoticed, and it is often accompanied by complications such as thrombophlebitis and sepsis [1-3]. The nature of GVT and its distinct characteristics were identified initially in a case report published in 1956 by Austin [4].

Almost 80% of GVT cases in the literature were reported for patients after delivery, hysterectomy, and lymphadenectomy for gynecological neoplasms [1,5-6]. GVT occurs most commonly (80%-90%) on the right [1-2,7]. In one study, the incidence of GVT was around 0.02% for vaginal deliveries and 0.1% for cesarean deliveries [8]. In the same study, 0.7% of twin deliveries via cesarean section were complicated by GVT, whereas no cases of GVT were observed for twin deliveries via the vaginal route.

The development of idiopathic GVT is even rarer, and we are not aware of its incidence being reported in the literature; it is mainly described in relation to pregnancy [6,8]. Pregnancy, pelvic inflammatory disease, pelvic surgery, and malignancy have been reported in association with the development of GVT [8-9]. The usual clinical presentation of GVT varies from asymptomatic to non-specific abdominal pain. Patients rarely present with acute abdominal pain [10].

Ultrasound, computed tomography (CT) imaging, and magnetic resonance imaging (MRI) can be used for the diagnosis of GVT. However, contrast-enhanced CT imaging of the abdomen and pelvis is the most reliable of these, with 100% sensitivity and 99% specificity [6]. The classical imaging findings are filling defects and enlargement of the gonadal vein [11]. Gonadal veins have extensive communication with the gonads and perineum, and this can facilitate the entry of infections [1].

In this study, we aimed to determine the 13-year incidence of GVT at a single tertiary hospital using CT imaging. We further describe GVT-associated risk factors.

## Materials and methods

A retrospective, single-center, observational study was conducted at King Abdulaziz Medical City in Jeddah, Saudi Arabia. The study was approved by the Institutional Review Board of King Abdullah International Medical Research Center. Data collection was performed by searching the center’s Radiology Information System for all patients diagnosed with incidental GVT using contrast-enhanced CT imaging of the abdomen and pelvis between January 2005 and December 2017. The search terms were “gonadal vein thrombosis” and “ovarian vein thrombosis.” The Hospital Information System and the Picture Archiving and Communication System were used to collect patient demographic profiles (namely, age and gender), clinical characteristics (namely, diagnosed diseases, comorbidities, previous surgeries, and malignancies), radiological features (namely, radiological modality, vein size, and thrombus size), and treatment parameters (namely, type of treatment, length of treatment, and outcome). All patients with an incidental finding of GVT were included, and those with incomplete data were excluded. Data were recorded in an encrypted Microsoft Excel (Microsoft Corporation, Redmond, WA) file for analysis.

All numerical values obtained were presented using simple descriptive statistics: means and standard deviations for continuous data, frequencies and percentages for categorical data. IBM SPSS Statistics for Windows version 21.0 (IBM Corp., Armonk, NY) was used for data analysis. All data collection sheets were anonymous, with no identifiers used. Privacy and confidentiality of information were completely maintained.

## Results

In total, 58 patients were diagnosed with incidental GVT using contrast-enhanced CT imaging during the study period, an overall incidence of 0.08%. Demographics, subject, and GVT characteristics are shown in Table 1. Fifty-seven patients (98%) were females and only one (2%) was male. The mean age (years) of the patients was 50.0±15.0 (range 4-87). Thirty-four patients (59%) had right GVT while 20 (34%) had left GVT. Only four patients (7%) had bilateral GVT. The mean diameter of the thrombosed vein was 10.09±3.56 mm (range 4.05-21.49 mm). Twenty-nine patients (50%) had acute thrombi at the time of imaging while the other 50% had chronic thrombi (i.e., acute thrombosis is considered whenever there is a central thrombus distending the lumen with surrounding contrast media; otherwise, chronic thrombosis is considered). All patients with bilateral GVT had chronic thrombi. Nineteen patients (33%) had concurrent thrombosis of another vein, most commonly the renal vein or the inferior vena cava. Seven patients (12%) had evidence of pulmonary embolism. Nineteen patients (33%) had undergone pelvic surgery previously. Forty-four patients (76%) had a malignancy at the time of diagnosis; of these, the most common sites affected were the breast (9 patients “20.5%”) and the colon (8 patients “18.2%”). Other sites included the uterus (4 patients “9.1%”), brain (3 patients “6.8%”), urinary bladder (3 patients “6.8%”), kidney (2 patients “4.5%”), lung (2 patients “4.5%”), small bowel (2 patients “4.5%”), shoulder (2 patients “4.5%”), ovaries (1 patient “2.3%”), stomach (1 patient “2.3%”), pancreas (1 patient “2.3%”), and gallbladder (1 patient “2.3%”), whereas other malignancies included lymphoma (2 patients “4.5%”), cholangiocarcinoma (1 patient “2.3%”), leukemia (1 patient “2.3%”), and Wilms (1 patient “2.3%”). Forty-two patients (72%) were treated with anticoagulants after the diagnosis of GVT while 16 (28%) were not treated at our center due to death prior to initiation of treatment, refusal to be treated, or preference to be treated at another hospital.

**Table 1:** Demographics, subject, and gonadal vein thrombosis (GVT) characteristics

Variable		Total Subject (n = 58)
Age in years “Mean (Standard deviation)”		50.0 (15.0)
Gender “n (%)”	Male	1 (2%)
	Female	57 (98%)
GVT side “n (%)”	Right	34 (59%)
	Left	20 (34%)
	Bilateral	4 (7%)
Thrombosed vein diameter in millimeter “Mean (Standard deviation)”		10.09 (3.56)
GVT acuity at the time of imaging “n (%)”	Acute	29 (50%)
	Chronic	29 (50%)
Concurrent thrombosis of another vein “n (%)”		19 (33%)
Evidence of pulmonary embolism “n (%)”		7 (12%)
Previous pelvic surgery “n (%)”		19 (33%)
Malignancy at the time of diagnosis “n (%)”		44 (76%)
Anticoagulants treatment “n (%)”	Yes	42 (72%)
	No	16 (28%)

## Discussion

The incidence of GVT from CT images in our study was 0.08%, with the right gonadal vein being involved in 59% of the patients diagnosed as such. The most common risk factor among the patients in our series was malignancy. The prevalence of malignancies among the patients is probably because ours is an oncology center. The increased susceptibility of the right side may be attributed to the longer length of the right gonadal vein than that of the left, and an increased absence of competent valves in the right compared to that in the left gonadal vein [5,12]. In addition, the antegrade blood flow in the right gonadal vein favors bacterial infection unlike the retrograde blood flow in the left gonadal vein [13]. Nevertheless, a higher incidence of thrombosis in the left than in the right ovarian vein was reported in one study [14]. Bilateral thrombi are observed in 11%-14% of cases [12-13]. In our study, the right gonadal vein was involved in 59% of patients, which is lower than the reported rate in the literature (i.e., 70%-90%), whereas the left gonadal vein was involved in more than 30% of patients, which is higher than the reported rate in the literature (i.e., 24%) [2,15]. Bilateral involvement was observed in 7% of patients, which is lower than the reported rate in the literature (i.e., 14%) [12-13,15].

Several dangerous complications may occur with GVT, including pulmonary embolism, sepsis with multiorgan failure, an extension of the thrombus into the inferior vena cava, iliac vein, or renal vein, and an increased risk of ovarian infarction [8-9]. Pulmonary embolism is an infrequent complication reported in the range of 13%-33% [8]. In our study, 33% of patients exhibited concurrent thrombosis of other veins at the time of diagnosis. In addition, 12% developed pulmonary embolism, which is in agreement with the reported rate in other studies [2,16].

Malignancy is a recognized prothrombotic condition with an incidence rate of as high as 20% in cases of venous thromboembolism [17]. Cancer is associated with changes in blood flow, damage to vascular endothelium, and changes in clotting factors [18]. Venous thromboembolism is associated with increased morbidity and considered a common cause of death in cancer patients [19]. Our results indicated that 76% of patients had a malignancy.

The management of GVT is mainly performed using anticoagulation therapy for three to six months. Extension of the thrombus to the renal vein and/or inferior vena cava is treated similar to pulmonary embolism [20]. Surgical management is reserved for patients with contraindications to anticoagulants and for those who develop pulmonary emboli despite conservative therapeutic management [21-22]. In our study, 72% of patients were successfully treated with anticoagulants. The remaining patients were not treated at our center due to death prior to the initiation of treatment, refusal to be treated, or preference to be treated at another hospital.

The study is limited due to its small sample size, retrospective nature, and all cases included were oncology cases from an oncology center. Yet, it showed that the incidence of GVT in oncology patients is similar to the overall population. Multicenter studies with a larger, more variable sample can be further assessed.

## Conclusions

This study revealed the incidence of GVT using CT scans and described its associated risk factors. GVT is a rare clinical entity with vague clinical presentation. Initiation of anticoagulation therapy is important to treat this condition and limit complications.
